# Supplementary material for: Speed dependent descending control of freezing behavior in Drosophila melanogaster
Source: Nat Commun. 2018 Sep 12;9:3697. doi: 10.1038/s41467-018-05875-1 (PMC6135764; doi:10.1038/s41467-018-05875-1)
Supplement: Supplementary file 1 — Supplementary Information [file 41467_2018_5875_MOESM1_ESM.pdf]

Supplementary Information

Supplementary Figures

Speed dependent descending control of freezing behavior in *Drosophila melanogaster*

Zacarias, et al.

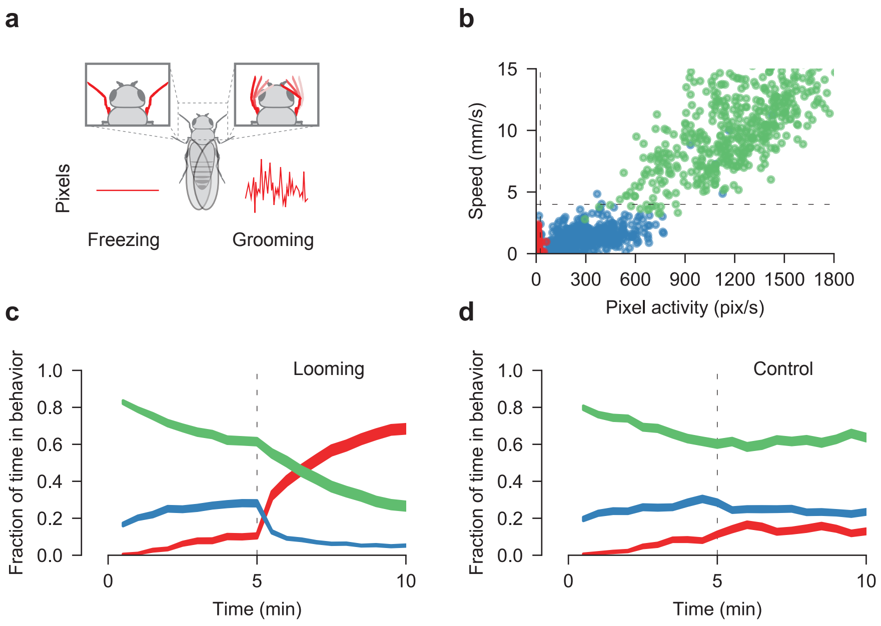

Supplementary Figure 1 – Classification of freezing, grooming and walking.
(a) Appendage movement during grooming induces pixel change whereas freezing does not (illustration by Gil Costa). **(b)** Scatter of speed and pixel change for manually scored bouts of walking, freezing and grooming (green, red and blue, respectively). Each data point corresponds to a 500 ms bin, n=500 for each behavior. Vertical and horizontal dashed lines indicate thresholds defined for classifying freezing (< 50 pix s^{-1}) and walking (> 4 mm s^{-1}), respectively. Events that are low speed but high pixel change correspond mostly to grooming. **(c,d)** Percentage of time spent performing each of the classified behaviors in 30 s bins (mean ± s.e.m.) for the looming (c) and control (d) conditions. Dashed line indicates onset of stimulation.

a

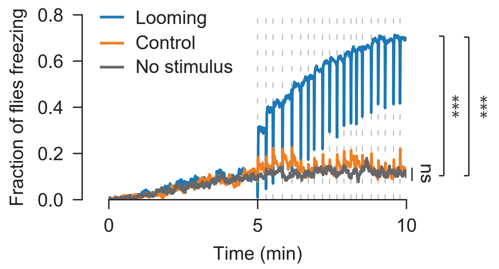

Supplementary Figure 2 - Flies freeze upon looming stimulation.
(a) Fraction of flies freezing. Dashed lines represent stimulus presentations. *** denotes p< 0.001 and ns=not significant, Chi-squared test.

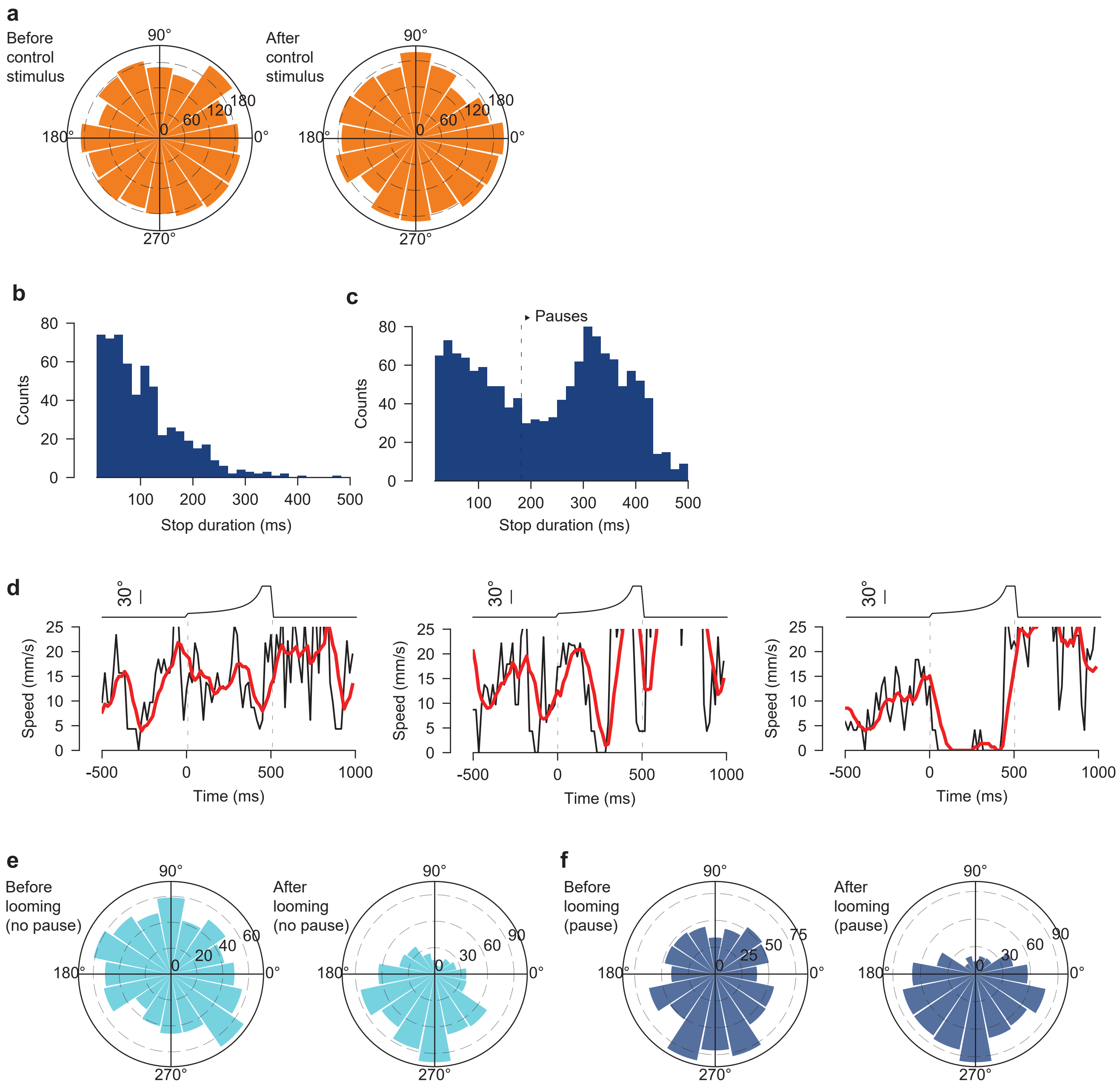

Supplementary Figure 3 – Fleeing events and pausing.
(a) Distribution of path orientations before (left) and after (right) control stimulus. Bar heights indicate counts. Stimulus source (screen) was located at 90º. **(b,c)** Distribution of stop durations during the 500 ms preceding looming (b, spontaneous pauses) and during looming presentation (c, looming-triggered pausing). The dashed line indicates the minimum duration threshold for classifying pauses. **(d)** Example speed traces for looming trials of walking flies (instantaneous speed, black; running average, red). No pause (left), short pause indistinguishable from spontaneous pauses (middle) and a looming-triggered pause (right). **(e,f)** Distribution of path orientations before (left) and after (right) looming stimuli for walking trials where flies did not pause to the stimulus (**e**, n=767) and trials where flies paused (**f**, n=806).

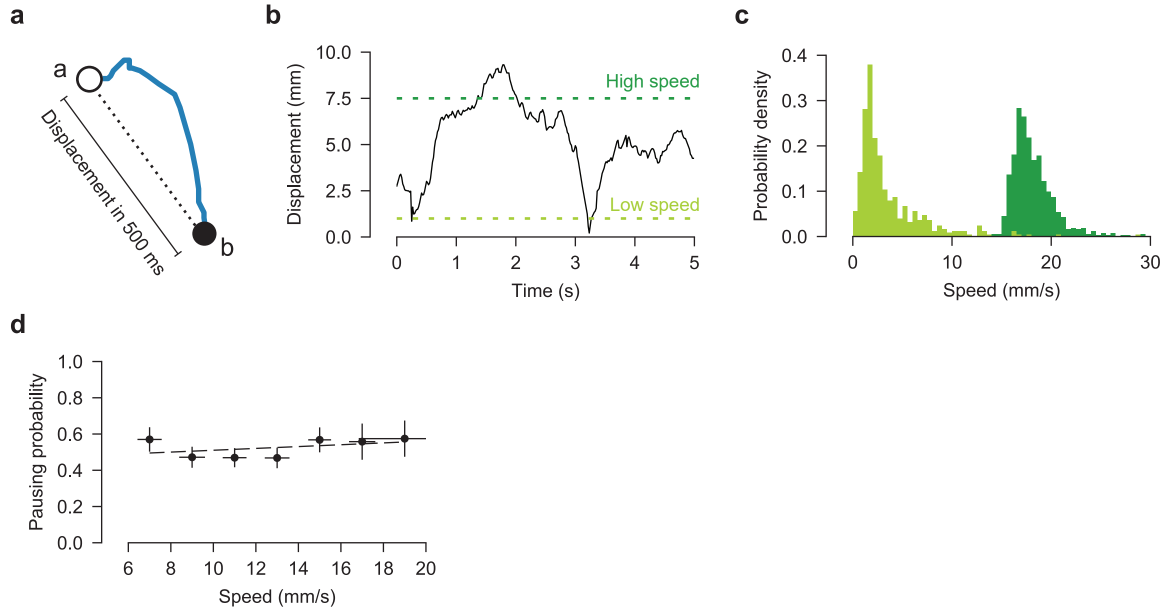

Supplementary Figure 4 – Closed-loop experiment.
(a) Example scheme of the displacement measure tracked in real time and used to trigger looming stimuli. The low speed group received looming when displacement was lower than 1 mm in the preceding 500 ms, and the high speed group when displacement was higher than 7,5 mm. In b,c dark and light green correspond to high and low speed groups, respectively. **(b)** Example trace of displacement over a 5 sec period. Dashed lines indicate threshold crossings for each condition. **(c)** Distribution of speed 500 ms before looming for low (n=526) and high speed (n= 895) conditions. Displacement generated two wide but unique distributions of pre-looming speed with very little overlap. **(d)** Probability of pausing as a function of pre-looming speed. X-error bars shows SD for speed sampled within each interval and Y-error bars show 95% confidence intervals (r^2 = 0.17, p=0.35).

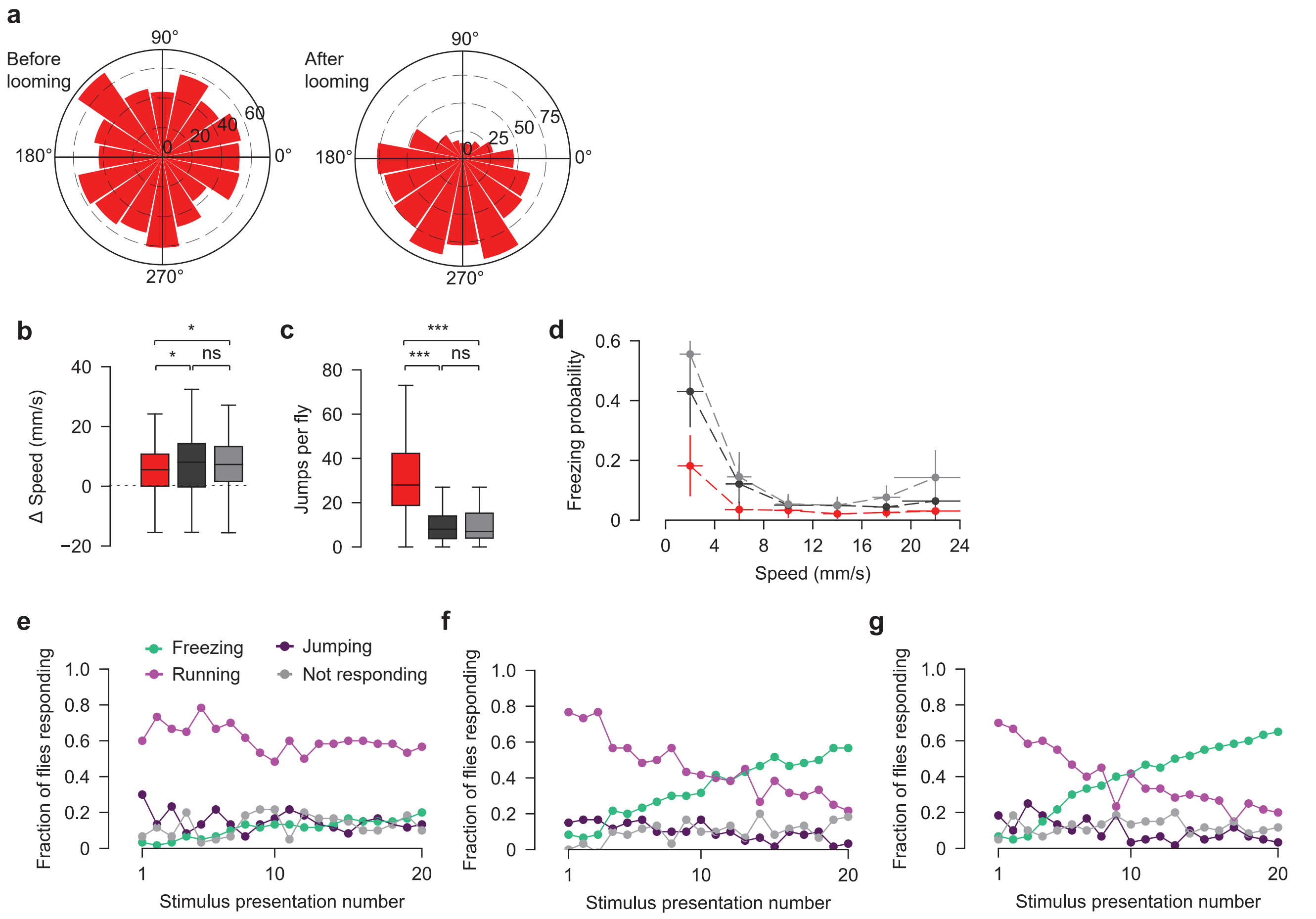

Supplementary Figure 5 – DNp09 neuron silencing.
In **a-d** red corresponds to DNp09>Kir2.1 flies, dark gray to DNp09/+ flies and light gray to Kir2.1/+ flies. **(a)** Distribution of path orientations of walking trials before (left) and after (right) looming for DNp09>Kir2.1 flies (n=822). Bar height indicates counts. Stimulus source (screen) was located at 90º (Wilcoxon signed-rank test, p<0.001). **(b)** Change in walking speed (pre-looming period subtracted from post-looming period) caused by stimulus presentation, including only walking flies that pause (DNp09>Kir2.1 n=234; DNp09/+ n=253, Kir2.1/+ n=259. p=0.01 Kruskal-Wallis and post-hoc Dunn tests). **(c)** Number of jumps per fly during the 5 min stimulation period (Kruskal-Wallis and post-hoc Dunn tests). **(d)** Probability of freezing to looming as a function of speed 500 ms before. X-error bars shows standard deviation for speed sampled within each interval and Y-error bars show 95% confidence intervals. **(e,f,g)** Fraction of flies performing the described behaviors for each of the 20 looming presentations. **(e)** DNp09>Kir2.1 **(f)** DNp09/+ **(g)** Kir2.1/+. * denotes p<0.05, *** p<0.001 and ns = not significant.

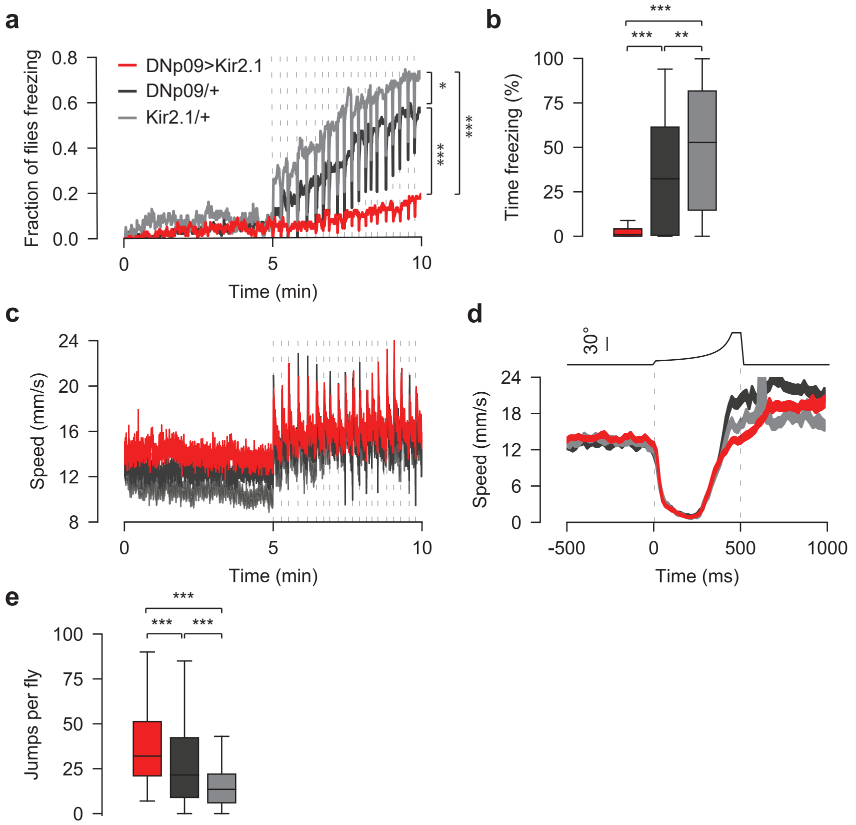

Supplementary Figure 6 – Silencing DNp09 neurons using wild-type DL to generate parental controls.

In **a-e** red corresponds to DNp09>Kir2.1 flies, dark gray to DNp09/+ flies and light gray to Kir2.1/+ flies. **(a)** Proportion of freezing flies (n=100 for all conditions). Dashed lines indicate stimulus presentations (Chi-squared test). **(b)** Percent time spent freezing during the 5 min stimulation period (Kruskal-Wallis and post-hoc Dunn tests). **(c)** Average (± SEM) fly speed including only time periods classified as walking. Dashed lines indicate stimulus presentations. **(d)** Looming-triggered speed profile (average ± SEM) including only walking trials that include a pause (DNp09>Kir2.1 n=451, DNp09/+ n=287, Kir2.1/+ n=223). **(e)** Number of jumps per fly during the stimulation period (Kruskal-Wallis and post-hoc Dunn tests). * denotes p<0.05, ** denotes p<0.01 and *** denotes p<0.001.
