# Supplementary material for: Speed dependent descending control of freezing behavior in Drosophila melanogaster
Source: Nat Commun. 2018 Sep 12;9:3697. doi: 10.1038/s41467-018-05875-1 (PMC6135764; doi:10.1038/s41467-018-05875-1)
Supplement: Supplementary file 3 — Description of Additional Supplementary Files [file 41467_2018_5875_MOESM3_ESM.pdf]

Description of Additional Supplementary Files:

Supplementary Movie 1 – Prolonged freezing in response to looming. Example video of a test session starting just prior to the first looming stimulus, until the end of the experiment (8x speed). Infra-red LED in the bottom left corner signals looming presentations. During the stimulation period, small movements (startle response) are apparent during each looming presentation (a salient example can be seen on second 29).

Supplementary Movie 2 – Freezing in grooming posture. Example of a fly exposed to a looming stimulus while grooming. Upon the looming stimulus, the fly freezes holding the grooming position for a long period of time (normal speed).

Supplementary Movie 3 – Fleeing response. Example of a running escape trial with a pause (normal speed).

Supplementary Movie 4 – Freezing upon optogenetic activation of DNp0P9 neurons. Example of a light stimulation event for flies expressing CsChrimson in DNp09 neurons. Infrared LED signals time of stimulation (normal speed).
